# Supplementary material for: Patient perspectives on patient similarity-based risk communication for uncontrolled type 2 diabetes in primary care: A qualitative study
Source: PLoS One. 2025 Jul 2;20(7):e0327623. doi: 10.1371/journal.pone.0327623 (PMC12221083; doi:10.1371/journal.pone.0327623)
Supplement: S1 Appendix — (PDF) [file pone.0327623.s001.pdf]

Patient similarity-based risk communication session

A. Opening

- Ask patient for his/her view on diabetes control (e.g. “How do you think you have fared in terms of controlling your diabetes?”)

B. Communication of results – glycaemic control

- HbA1c
- HbA1c in relation to target – well-controlled (within HbA1c target), not well-controlled (< 1.0% above target) or poorly controlled (≥ 1.0% above target)
- Explain relation of HbA1c and glucose
- Explain HbA1c ranking among ‘peers’ (i.e. ‘people like you’)
- Ask patient if he/she has any explanation for the results (e.g. missing medication doses accounting for the HbA1c increase)
- Ask patient to share what he/she thinks of the result

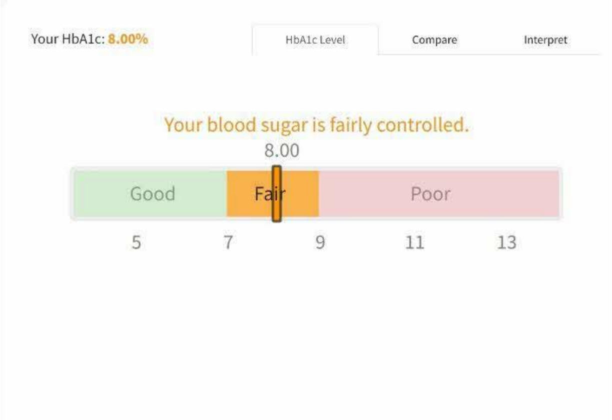

Fig 1. HBA1c in relation to individual target

HbA1c shows your average diabetes control since the last visit
<u>Lower</u> HbA1c means <u>better</u> diabetes control
<u>Higher</u> HbA1c means <u>worse</u> diabetes control

For people without diabetes, HbA1c is usually 4-6%
For people with diabetes, HbA1c below 7% means the diabetes is well controlled

Your HbA1c is 8.0%
This is above the target level of < 7%

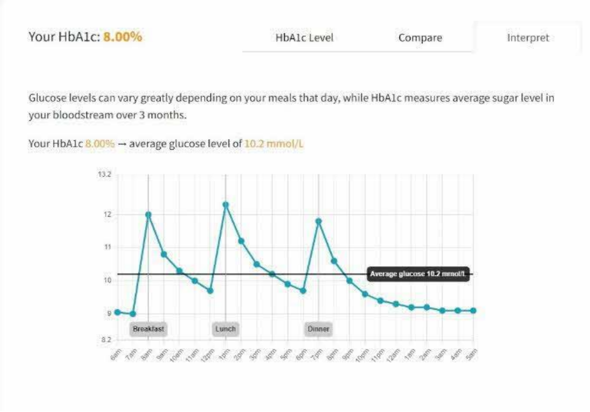

Fig 2. Relation of HbA1c and glucose

HbA1c is different from glucose
Whether you fasted or ate on the day of your test, this does not affect the HbA1c reading

For reference, to achieve an average glucose of below 7, you need HbA1c below 6%

Your HbA1c of 8.0% means that your average glucose is 10.2
This is considered to be very high
It also accounts for fluctuating glucose levels throughout the day which are not measured unless your check them at home

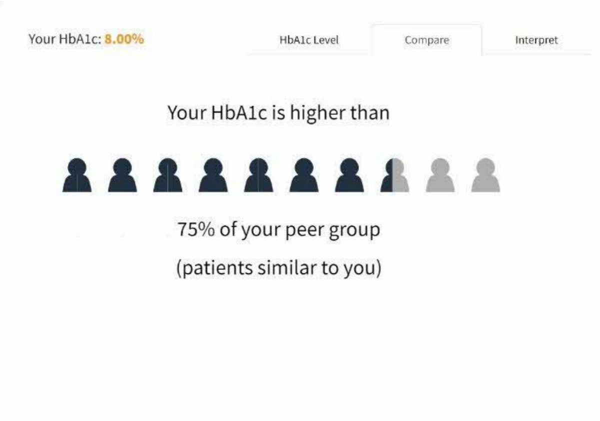

Fig 3. HbA1c ranking among similar patient cohort ('peers')

There are 'people like you' who are similar in age, diabetes duration, body mass index, blood pressure, cholesterol levels and medications

Among 10 such people, your HbA1c is worse than 7 of them
This means 7 in 10 people have better diabetes control than you

C. Communication of risk – potential complications

- What may happen
- Why/how it may happen
- How patient may be affected (i.e. how patient may experience the complications)
- % of 'peers' (i.e. 'people like you') with diabetes complications (i.e. complication prevalence) – comparing those with poorer glycaemic control (higher HbA1c) and those with better glycaemic control (lower HbA1c)

- Compare ‘poor control’ and ‘good control’ patient examples in terms of HbA1c trajectory and complications developed
- Ask patient to share what he/she thinks of the risks

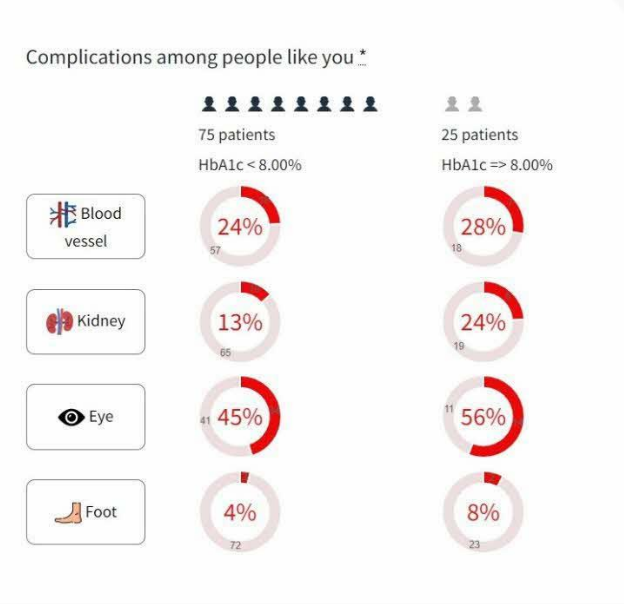

Fig 4. Complication prevalence % among the similar patient cohort

Many complications can result from diabetes when it is uncontrolled

For example

- Blood vessel blockage – leading to stroke and heart attack
- Kidney disease – leading to kidney failure and dialysis
- Eye disease – leading to blindness
- Foot problems such as nerve damage and losing sensation of your feet – leading to leg wounds and amputation

Here are 100 ‘people like you’ who are similar in age, diabetes duration, body mass index, blood pressure, cholesterol levels and medications

You can see that overall, those with better HbA1c than you (HbA1c below 8.0%) have fewer complications from diabetes

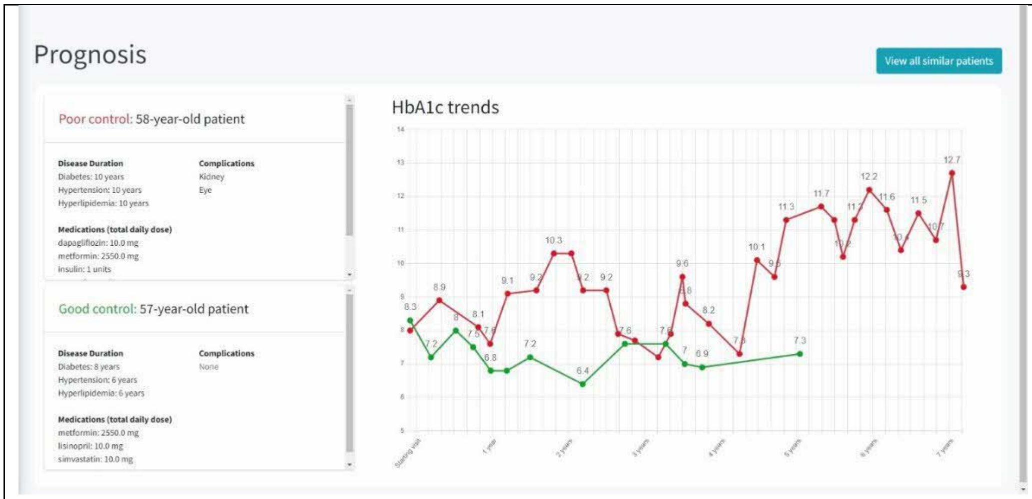

Fig 5. Case examples from the similar patient cohort

Let's look at two patient examples:

1. Poor control
- This person's HbA1c worsened over time / fluctuated a lot
- He/she developed more complications / developed complications earlier on in the course of his/her diabetes

2. Good control
- This person's HbA1c improved over time / was maintained
- He/she developed fewer complications / the onset of complications was delayed

It is common to feel completely normal even when your diabetes is poorly controlled
But in reality, you can see that i) most people's diabetes control is better than yours, and ii) many people like you already developed complications from diabetes
It is important to take steps now to control your diabetes, to achieve the best possible outcome for your health and well-being

D. Recommendations
- Ways (i.e. health actions) to improve glycaemic control and/or prevent complications (or further complications)
  - medication adjustment
  - medication adherence
  - diabetes self-care activities – diet, exercise, blood glucose monitoring, foot care, etc.
- Ask patient to share what he/she thinks of the recommendations
