# Supplementary material for: Patient perspectives on patient similarity-based risk communication for uncontrolled type 2 diabetes in primary care: A qualitative study
Source: PLoS One. 2025 Jul 2;20(7):e0327623. doi: 10.1371/journal.pone.0327623 (PMC12221083; doi:10.1371/journal.pone.0327623)
Supplement: S2 Appendix — AI digital tool. This is a detailed description of the modules in the PERDICT.AI digital tool. (PDF) [file pone.0327623.s002.pdf]

**PERDICT.AI digital tool**

| Module | Description |
|---|---|
| 1: HBA1c control and comparison against a similar patient cohort[a] ("Peer comparison") | Patient's HBA1c is placed into a colour-coded category of control (individual-level HBA1c control). The HBA1c is ranked amongst a cohort of patients with similar clinical and demographic features (similar patient cohort) at the primary care institution, identified using a K-nearest neighbour similarity algorithm [32,33]. |
| 2A: Complications among the similar patient cohort | Patient is shown the % prevalence of diabetes complications among a similar patient cohort. This can be further subcategorised into two subgroups based on HBA1c and the complication prevalence compared between the subgroups, to allow loss-framing and gain-framing. |
| 2B: Case narrative of similar patients | Similar patient case examples of suboptimal control (i.e. suboptimal diabetes control and more complications developed) and good control (i.e. good diabetes control and fewer complications developed) are illustrated to the patient. |
| 3: Medication recommender | Diabetes medications are recommended according to a rule-based algorithm in accordance with local clinical practice guidelines. Information is provided on the expected HBA1c effect, cost and side effects of each medication; can be compared with alternative medications. |
| 4: Care plan | Doctor and patient co-create a personalised care plan with action measures (diet, exercise, medications and monitoring plans) and setting of targets (HBA1c, weight). |

[a]Based on de-identified data from electronic medical records over a 10-year period (1 April 2012 to 31 March 2022), accessed between 2 Aug 2023 and 28 Aug 2023
