# Supplementary material for: Patient perspectives on patient similarity-based risk communication for uncontrolled type 2 diabetes in primary care: A qualitative study
Source: PLoS One. 2025 Jul 2;20(7):e0327623. doi: 10.1371/journal.pone.0327623 (PMC12221083; doi:10.1371/journal.pone.0327623)
Supplement: S3 Appendix — (PDF) [file pone.0327623.s003.pdf]

Interview topic guide

Section	Topic	Example question(s)
Opening	Overall experience	How did the session go for you?
Social comparison orientation	Preference for/against social comparison	How did you feel when we compare your diabetes to other people? Should we be comparing your diabetes to other people? Which groups of people should we use for comparison?<br><br>In the patient examples, can you see a snapshot of yourself (present/future)?<br><br>Do you think you are similar in profile to the examples we used? In what way are they similar / not similar?<br><br>Is it helpful to know how people like yourself have fared in controlling their diabetes?<br><br>Is it helpful to know how other people like yourself have fared in preventing diabetes complications?
Perceptions	Effect of social comparison on risk (threat) perceptions<br>- perceived likelihood of diabetes complications<br>- perceived severity of diabetes complications	Did you learn anything new about your risk of getting complications from diabetes? Which part(s) of the session helped with this? Is there a better way we can help you understand this?<br><br>Did you learn anything new about the complications of diabetes? Which part(s) of the session helped with this? Is there a better way we can help you understand this?<br><br>Do numbers/percentages help you understand risk?<br><br>What (what else) comes to your mind when thinking about your risk of getting complications from diabetes?
Perceptions	Expectations of recommended advice<br>- perceived benefits<br>- perceived barriers	Are you convinced that the recommended advice would improve your diabetes?<br><br>What would hinder you from following through with the recommended advice?
Action	Cues to action	What triggers you to think about doing something to improve your diabetes?
Action	Action uptake	What would push you into following the recommended advice to improve your diabetes?
