# Supplementary material for: Patient perspectives on patient similarity-based risk communication for uncontrolled type 2 diabetes in primary care: A qualitative study
Source: PLoS One. 2025 Jul 2;20(7):e0327623. doi: 10.1371/journal.pone.0327623 (PMC12221083; doi:10.1371/journal.pone.0327623)
Supplement: Attachment — Submitted filename: PlosOne PRICOM - Response to Reviewers 20250509.docx [file pone.0327623.s005.docx]

09 May 2025

Academic Editor

PLOS ONE

Dear Editor and Reviewers,

**Re: Response to reviewers’ comments on PONE-D-24-37711**

Thank you for giving us the opportunity to improve our manuscript. We appreciate the time and effort you have taken to provide valuable feedback and have revised our manuscript.

We have provided our written responses to each of the points raised in your earlier letter.

**EDITOR’S COMMENTS**

1. Journal Requirements:

Please review your reference list to ensure that it is complete and correct. If you have cited papers that have been retracted, please include the rationale for doing so in the manuscript text, or remove these references and replace them with relevant current references. Any changes to the reference list should be mentioned in the rebuttal letter that accompanies your revised manuscript. If you need to cite a retracted article, indicate the article’s retracted status in the References list and also include a citation and full reference for the retraction notice.

**Response:**

Thank you for highlighting this. The following changes have been made to the reference list in our revised manuscript:

**a) Added new references #26, #27, #30, #31, #32, #35 and #42:**

26. Sharafoddini A, Dubin JA, Lee J. Patient Similarity in Prediction Models Based on Health Data: A Scoping Review. JMIR Med Inform. 2017;5(1):e7. Published 2017 Mar 3. doi:10.2196/medinform.6730

27. Parimbelli E, Marini S, Sacchi L, Bellazzi R. Patient similarity for precision medicine: A systematic review. J Biomed Inform. 2018;83:87-96. doi:10.1016/j.jbi.2018.06.001

30. Ong R, Ng CJ, Gunasekaran K, et al. Utility of a patient similarity-based digital tool for risk communication to patients with type 2 diabetes mellitus: perspectives from primary care physicians in ambulatory care. PLoS One. 2025;20(3):e0319992. doi:10.1371/journal.pone.0319992

31. Beeney LJ, Fynes-Clinton EJ. The Language of Diabetes Complications: Communication and Framing of Risk Messages in North American and Australasian Diabetes-Specific Media. Clin Diabetes. 2019;37(2):116-123. doi:10.2337/cd18-0024

32. Lim WM. What is qualitative research? An overview and guidelines. Australasian Marketing Journal (AMJ). 2024;0(0). doi:10.1177/14413582241264619

35. Hennink M, Kaiser BN. Sample sizes for saturation in qualitative research: A systematic review of empirical tests. Soc Sci Med. 2022;292:114523. doi: 10.1016/j.socscimed.2021.114523. Epub 2021 Nov 2. PMID: 34785096.

42. Lipsey AF, Waterman AD, Wood EH, Balliet W. Evaluation of first-person storytelling on changing health-related attitudes, knowledge, behaviors, and outcomes: A scoping review. Patient Educ Couns. 2020;103(10):1922-34. doi:10.1016/j.pec.2020.04.014

**b) Removed references #15-19 and #21**

**c) Renumbered the following references:**

| Previous # | Renumbered as |
| --- | --- |
| #20 | #23 |
| #22 | #24 |
| #23 | #25 |
| #24 | #19 |
| #25 | #20 |
| #26 | #21 |
| #27 | #22 |
| #28 | #18 |
| #29 | #15 |
| #30 | #16 |
| #31 | #17 |
| #32 | #28 |
| #33 | #29 |
| #35 | #36 |
| #36 | #37 |
| #37 | #38 |
| #38 | #39 |
| #40 | #41 |
| #41 | #33 |
| #42 | #43 |
| #44 | #45 |
| #46 | #47 |
| #47 | #48 |

**d) References 1-14 and 34 remain unchanged**

2. Additional Editor Comments:

The manuscript has a good deal of merit for publication after minor revisions.

Given the similarities between the two articles, we request the authors to provide a clear justification for the need to present these findings separately. Alternatively, they may consider combining their results into a single, comprehensive manuscript. This approach could strengthen the impact of their study by providing a more holistic perspective on the research question.

**Response:**

Thank you for your feedback. The previous study focused on primary care physicians who are the main stakeholders to communicate diabetes-related risks to patients using PERDICT.AI. The key themes from that study were ‘Education and motivation for subgroups of patients with T2DM’, ‘Patients who do not practise social comparison’, and ‘Potential for false reassurance or negative reactions from patients’. The findings informed us to narrow down the target recipients of the risk communication to patients with uncontrolled T2DM, instead of all T2DM patients regardless of glycaemic control. Therefore, this study explored the perspectives and experiences of this patient subgroup on how their diabetes-related risks were communicated to them by the physician using PERDICT.AI. The key themes were ‘I am myself’, ‘Motivation to be like the majority’, ‘Avoiding similar negative outcomes’, ‘Low risk does not equate to zero risk’, and ‘Motivation to replicate how others achieve positive outcomes’. Thus, the perspectives from these two major stakeholders are presented in two separate manuscripts.

We have added the following sentences (changes in bold) to the ‘Introduction’ section:

*‘PERDICT.AI (Personalised Diabetes Counselling Tool using Artificial Intelligence), a digital tool based on an AI-driven patient similarity model, was developed by a team of primary care physicians (PCPs) and data scientists to support PCPs in patient similarity-based risk communication during their consultations with patients who have type 2 diabetes mellitus (T2DM) [28,29].* ***Based on the pilot study involving PCPs [30], risk communication leveraging on PERDICT.AI is likely to be more effective for patients with uncontrolled T2DM and who are receptive to social comparison. Furthermore, PCPs alluded to patients being discouraged or distressed when their glycaemic control is compared to that of other patients, particularly when their glycaemic control is due to circumstances out of their control. This is an unintended consequence of risk communication about diabetes complications that could interfere with diabetes self-management [31]. The findings informed us to narrow down the target recipients of the risk communication to patients with uncontrolled T2DM, instead of all T2DM patients regardless of glycaemic control. Therefore, this study explored the perspectives and experiences of this patient subgroup on how their diabetes-related risks were communicated to them by a PCP using PERDICT.AI. The findings will allow comparison of perspectives from these two major stakeholders on patient similarity-based risk communication using PERDICT.AI.’***

The manuscript of the previous study with physician participants is cited as reference #30 in the revised manuscript. We have uploaded a copy named ‘journal.pone.0319992.pdf’, which replaces the old file ‘PONE-D-24-29527.pdf’.

References #30 and #31 are newly cited in the revised manuscript.

3. Comments from PLOS Editorial Office: We note that one or more reviewers has recommended that you cite specific previously published works. As always, we recommend that you please review and evaluate the requested works to determine whether they are relevant and should be cited. It is not a requirement to cite these works. We appreciate your attention to this request.

**Response:**

Thank you for highlighting this. We have read the articles recommended by the reviewers and have decided not to cite them. Do refer to our replies to the reviewer comments.

**REVIEWERS’ COMMENTS**

**Reviewer #1:**

1. Title: Appropriate and informative.

**Response:**

Thank you for your feedback.

2. Abstract: Appropriate and informative.

Number of words: 320 words (Maximum 300 words; not correct according to the instructions of the authors).

**Response:**

Thank you for your feedback. We have shortened the Abstract.

3. Keywords: Please use/add specific MeSH words like; Qualitative analysis, Type 2 diabetes mellitus, Singapore, PERDICT.AI.

**Response:**

Thank you for your feedback. We have revised the keywords to the following MeSH terms:

Diabetes Mellitus, Type 2

General Practice

Singapore

Digital Health

Qualitative Research

4. Introduction: Appropriate and informative.

**Response:**

Thank you for your feedback.

5. Objectives: Appropriate and informative.

**Response:**

Thank you for your feedback.

6. Methods:

The title of the section could be changed to be “Methodology” OR “Subjects and Methods” OR “Patients and Methods”.

Appropriate and informative.

**Response:**

Thank you for your feedback. We have renamed the section title to ***‘Methodology’.***

7. Statistical Analysis: Appropriate and informative; needs more details.

**Response:**

Thank you for your feedback. The 'Data analysis' section now reads as follows (changes in bold):

*‘All audio recordings were transcribed verbatim and checked for completeness and accuracy. RO and KG familiarised themselves with the transcripts from the first two in-depth interviews and independently coded the transcripts. Open codes were assigned to the transcripts based on the study objective, and were combined to form a coding framework.* ***RO and KG met to discuss and reach a consensus on shared meanings of the codes, and discrepancies were resolved through consultation with an experienced qualitative researcher CJN.*** *RO coded the remaining transcripts and new codes were added iteratively after discussion with the study team. The data was managed using NVivo Windows Release 1.5.1. Emergent themes were identified.* ***Thematic saturation was reached after 12 interviews. No new themes emerged from the remaining interviews.****’*

8. Results:

Text of the results: Appropriate and informative; but long.

Please do not repeat in the text what has been presented in the tables.

**Response:**

Thank you for your feedback. We have removed the repeated text, and also detailed the reasons for participant dropout (changes in bold) in the ‘Participant characteristics’ sub-section of the ‘Results’ section:

***‘18 of the 20*** *participants in the intervention arm completed the patient similarity-based risk communication session and interview.* ***Among the two participants who withdrew before the session, one cited loss of interest in the study and another cited ill health after unsuccessful rescheduling of the session. Characteristics of participants are detailed in Table 1.’***

9. Discussion: Appropriate and informative, however it needs to discuss with more details every finding of the study.

**Response:**

Thank you for your feedback. We have added the following sentences and paragraphs (changes in bold) to the ‘Discussion’ session:

***‘The study revealed diverse risk perceptions among the patient participants. Several of them were motivated to improve their glycaemic control to be compatible with most local patients with T2DM. In contrast, a few were resigned to be among the minority at risks of complications. The findings are consistent with PCPs’ perspectives from the previous study that risk communication using PERDICT.AI would more likely benefit patients who favour social comparison. While PCPs in the previous study had perceived that patients could potentially misinterpret or misunderstood their risks in using the PERDICT.AI [30], none of the patients in the present study alluded to such risk when they were compared with other “similar” patients.***

***With fear and risk aversity elicited among some participants, the study team recognises the potential for patient similarity-based risk communication to cause increased anxiety, frustration, and possibly disengagement in certain patients. This is consistent with the concerns PCPs raised in the previous study [30] and is a known unintended consequence of risk communication about diabetes complications that could interfere with diabetes self-management [31]. These occurrences can be minimised by PCPs’ awareness of their patients’ contextual factors contributing to glycaemic control, anticipation of negative reactions, and ability to frame and communicate risk information in a person-centric manner while taking the opportunity to motivate patients to improve their glycaemic control.***

*The findings are consistent with known literature on upward social comparison’s ability to engage competitiveness and facilitate information seeking to improve one’s health [18]. They are also consistent with known literature on risk perceptions having both “deliberative” (rule-based using absolute or comparative numerical information) and “affective” contextual factors that drive behavioural decisions [14,36-40], as demonstrated by participants who were motivated to reduce their complication risk to zero due to fear of being like the cohort minority who developed complications (i.e. “affective” risk perception), despite the minority being numerically small (i.e. not applying numerical principals seen in “deliberative” risk perception) [41].*

***Some participants wanted to know how to improve their glycaemic control and lower their risk of diabetes complications, seeking to replicate methods which worked for other patients. Future versions of PERDICT.AI should consider incorporating data on lifestyle measures. Patient narratives, through sharing of personal stories, can be used in tandem to motivate uptake of protective health behaviours and reduce fears among similar patients [42].***

***Patient similarity-based risk communication should be targeted at patients who are receptive to social comparison. Patients without such inclination may benefit from other modes of counselling to raise their risk awareness. Nuances in social comparison practices among the participants*** *shows that self-reported information on social comparison tendencies alone do not reliably predict whether a patient would be receptive to patient similarity-based risk communication.* ***Therefore,*** *challenges are anticipated in identifying and stratifying the nuanced layers of social comparison practices among patients using existing questionnaires such as INCOM (Iowa-Netherlands Comparison Orientation Measure) [33].* ***Further research is needed to operationalise the stratification of T2DM patients based on their social comparison tendencies and to develop validated measurement tools for data collection. For example, a potential direction is to prospectively study social comparison occurrences, characteristics, and its consequences on T2DM patients practising behavioural change using ‘ecological momentary assessment’ after participants are trained to recognise day-to-day social comparison occurrences. This method intensely captures the nuances of time-sensitive within-person social comparison occurrences and effects over multiple short time periods throughout the day and is less prone to recall bias compared to retrospective self-reporting methods [43]. The findings would shed new insight on how to identify patients who are most*** *receptive to patient similarity-based risk communication and to prioritise its use for these patients, with the aim of advancing their risk awareness and motivating the adoption of protective health behaviours [44]. The effectiveness of the risk communication would also depend on the PCP’s ability during a doctor-patient dialogue to frame the information in a person-centered manner, while avoiding the tendency to “climb probability trees” [45,46].*

***The study has limitations. Convenience sampling, rather than purposive sampling, was used due to challenges in recruiting patients into this study, introducing selection bias and limiting generalisability. Relevant attributes such as ethnicity and health literacy may not be adequately represented. Nevertheless, there was a good representation of the participants in terms of age, gender, education level, diabetes duration and control, which provided some degree of variation.*** *The complication prevalence among the SPC should not be interpreted as an individual’s predicted risk of developing complications. For such predictions, validated risk calculators should be used, such as the UK Prospective Diabetes Study (UKPDS) [47] in the UK and Framingham Adult Treatment Panel ATP III [48] in Singapore for cardiovascular disease risk calculation.****’***

References #30, #31 and #42 are newly cited in the revised manuscript.

10. The Limitations Section of the study; more possible biases?

**Response:**

Thank you for highlighting this. We have described further limitations and biases (changes in bold) in the ‘Discussion’ section:

***‘The study has limitations. Convenience sampling, rather than purposive sampling, was used due to challenges in recruiting patients into this study, introducing selection bias and limiting generalisability. Relevant attributes such as ethnicity and health literacy may not be adequately represented. Nevertheless, there was a good representation of the participants in terms of age, gender, education level, diabetes duration and control, which provided some degree of variation.*** *The complication prevalence among the SPC should not be interpreted as an individual’s predicted risk of developing complications. For such predictions, validated risk calculators should be used, such as the UK Prospective Diabetes Study (UKPDS) [47] in the UK and Framingham Adult Treatment Panel ATP III [48] in Singapore for cardiovascular disease risk calculation.’*

11. Conclusion: Conclusions need to be more concise and specific.

**Response:**

Thank you for your feedback. We have rewritten the ‘Conclusion’ section and it now reads:

***‘******This study provided insights into the acceptability of using a social comparison approach in communicating risk to patients with uncontrolled T2DM. It highlights the importance of identifying and selecting patients who are receptive to social comparison, clarifying patients’ perceptions of risks, including zero risk, and providing tailored and socially comparable strategies to mitigate these risks.’***

12. References:

Most references are old (>50% of the study references were published before 2019).

Please use Vancouver style in all references.

**Response:**

Thank you for your feedback. We have replaced the old references #15-19 and #21 with 6 new references (#26, #27, #30, #31, #32, #35 and #42), of which 5 of these are published on/after 2019.

The references are as follows:

26. Sharafoddini A, Dubin JA, Lee J. Patient Similarity in Prediction Models Based on Health Data: A Scoping Review. JMIR Med Inform. 2017;5(1):e7. Published 2017 Mar 3. doi:10.2196/medinform.6730

27. Parimbelli E, Marini S, Sacchi L, Bellazzi R. Patient similarity for precision medicine: A systematic review. J Biomed Inform. 2018;83:87-96. doi:10.1016/j.jbi.2018.06.001

30. Ong R, Ng CJ, Gunasekaran K, et al. Utility of a patient similarity-based digital tool for risk communication to patients with type 2 diabetes mellitus: perspectives from primary care physicians in ambulatory care. PLoS One. 2025;20(3):e0319992. doi:10.1371/journal.pone.0319992

31. Beeney LJ, Fynes-Clinton EJ. The Language of Diabetes Complications: Communication and Framing of Risk Messages in North American and Australasian Diabetes-Specific Media. Clin Diabetes. 2019;37(2):116-123. doi:10.2337/cd18-0024

32. Lim WM. What is qualitative research? An overview and guidelines. Australasian Marketing Journal (AMJ). 2024;0(0). doi:10.1177/14413582241264619

35. Hennink M, Kaiser BN. Sample sizes for saturation in qualitative research: A systematic review of empirical tests. Soc Sci Med. 2022;292:114523. doi: 10.1016/j.socscimed.2021.114523. Epub 2021 Nov 2. PMID: 34785096.

42. Lipsey AF, Waterman AD, Wood EH, Balliet W. Evaluation of first-person storytelling on changing health-related attitudes, knowledge, behaviors, and outcomes: A scoping review. Patient Educ Couns. 2020;103(10):1922-34. doi:10.1016/j.pec.2020.04.014

The new references are cited in the clean copy of the revised manuscript in line 64 (#26, #27), line 67 (#27), line 68 (#26), line 74 (#30), line 79 (#31), line 90 (#32), line 112 (#35), lines 336 and 341 (#30), line 342 (#31), and line 358 (#42). Despite being published before 2019, we cited #26 and #27 for being the most recent scoping/systemic reviews available on patient similarity models.

13. S1 Table: It is repeated.

**Response:**

Thank you for highlighting this. We have moved this to the ‘Supporting information’ section and renamed it ***‘S1 Appendix’***.

**Reviewer #2:**

1. The document is well written with only one typographical error identified in the data analysis section which should read: 'RO, KG and CJN met 'to reach' a consensus on the coding framework. instead of 'RO, KG and CJN met 'to reached' a consensus on the coding framework'.

**Response:**

Thank you for highlighting this. The error has been corrected. The revised sentence is as follows:

***‘RO and KG met to discuss and reach a consensus on shared meanings of the codes, and discrepancies were resolved through consultation with an experienced qualitative researcher CJN.’***

**Reviewer #3:**

Respected Editor,

Thank you for the opportunity to review the manuscript "Utility of Patient Similarity-Based Risk Communication During Primary Care Consultations: Perspectives from Patients with Uncontrolled Type-2 Diabetes." This study explores how patient similarity-based risk communication is perceived by individuals with uncontrolled type 2 diabetes in primary care settings. The research assesses its feasibility and potential impact on patient engagement, decision-making, and diabetes management.

I recommend the following major revisions in the manuscript:

1. The title is informative but making it more specific would help highlight the qualitative nature of the study. The term "utility" is somewhat vague; specifying whether it refers to feasibility, acceptability, or effectiveness could make it clearer. It would be helpful to revise the title to something like “Patient Perspectives on Patient Similarity-Based Risk Communication for Uncontrolled Type 2 Diabetes: A Qualitative Study” to explicitly reflect the study design.

**Response:**

Thank you for your feedback. We have revised the manuscript title to ***“Patient perspectives on patient similarity-based risk communication for uncontrolled type 2 diabetes in primary care: A qualitative study”***.

2. In the introduction, the background is well-structured, but it primarily focuses on the rationale for risk communication. While this is important, it would be beneficial to also discuss the possible limitations of social comparison-based approaches. Addressing potential drawbacks, such as patient distress from unfavourable comparisons or unintended negative effects on motivation, would provide a more balanced perspective.

**Response:**

Thank you for your feedback. We have included a description on the unintended negative effects of social comparison-based approaches in the ‘Introduction’ section:

***‘Furthermore, PCPs alluded to patients being discouraged or distressed when their glycaemic control is compared to that of other patients, particularly when their glycaemic control is due to circumstances out of their control. This is an unintended consequence of risk communication about diabetes complications that could interfere with diabetes self-management [31].’***

Reference #31 is newly cited in the revised manuscript.

3. The introduction does not clearly state what is already known about patient similarity-based risk communication and the specific gaps this study aims to fill. It would strengthen the introduction to explicitly highlight what makes this study novel by contrasting it with previous research.

**Response:**

Thank you for your feedback. We have added the following sentences and paragraphs (changes in bold) to the ‘Introduction’ section:

*‘Diabetes carries a huge economic burden in Singapore and around the world [1-3]. Among the diabetic population in Singapore, younger adults tend to have poorer glycaemic control and medication adherence [4-6]. The inertia to adopt protective health behaviours is contributed by knowledge gaps about diabetes and its complications, resulting in underestimation of the risks of diabetes complications [7-11]. To drive intention and uptake of protective health behaviours, it is pertinent that patients with diabetes have accurate risk perceptions about their health conditions [12-14].*

***A potential strategy to increase risk perception and motivate the adoption of protective health behaviours is to leverage on social comparison for risk communication [15-17], by means of comparing a diabetic patient’s glycaemic control and disease trajectory to other patients of similar clinical and demographic profiles [18-22]. Patient similarity-based models are shown to more effectively predict chronic disease outcomes compared to general population-based models [23-27], underpinned by the concept that “similar patients with similar features have similar outcomes” [25]. This is especially prevalent in cancer research where genetic or molecular profiles are used to predict the effectiveness of cancer treatments [25,27]. In the context of diabetes however, patient similarity-based models tend to focus on predicting diabetes onset [24,26] or effectiveness of pharmacological treatment [23], rather than predicting the complications arising from diabetes.***

*PERDICT.AI (Personalised Diabetes Counselling Tool using Artificial Intelligence), a digital tool based on an AI-driven patient similarity model, was developed by a team of primary care physicians (PCPs) and data scientists to support PCPs in patient similarity-based risk communication during their consultations with patients who have type 2 diabetes mellitus (T2DM) [28,29].* ***Based on the pilot study involving PCPs [30], risk communication leveraging on PERDICT.AI is likely to be more effective for patients with uncontrolled T2DM and who are receptive to social comparison. Furthermore, PCPs alluded to patients being discouraged or distressed when their glycaemic control is compared to that of other patients, particularly when their glycaemic control is due to circumstances out of their control. This is an unintended consequence of risk communication about diabetes complications that could interfere with diabetes self-management [31]. The findings informed us to narrow down the target recipients of the risk communication to patients with uncontrolled T2DM, instead of all T2DM patients regardless of glycaemic control. Therefore, this study explored the perspectives and experiences of this patient subgroup on how their diabetes-related risks were communicated to them by a PCP using PERDICT.AI. The findings will allow comparison of perspectives from these two major stakeholders on patient similarity-based risk communication using PERDICT.AI.’***

References #26, #27, #30 and #31 are newly cited in the revised manuscript.

4. In the methods, the study design is qualitative, but the justification for choosing qualitative methods is not provided. It would be helpful to explain why this approach was preferred over a mixed-methods design. Clearly stating why qualitative interviews were the most suitable method would improve the clarity of the methodology.

You may consider citing the following study:

DOI: 10.2147/RMHP.S296113

This study provides a conceptual framework and uses qualitative methods to explore collaborative medication therapy management. It can help justify why qualitative research is an appropriate approach in healthcare communication studies.

(This is optional and should only be taken as a suggestion for the improvement of the manuscript.)

**Response:**

Thank you for your feedback. We have added the following sentences to the ‘Study design and setting’ sub-section of the ‘Methodology’ section to explain the rationale for choosing a qualitative design:

***‘******A qualitative methodology was chosen to explore how patients responded to the risk information PCPs provided to them using PERDICT.AI. This included the complexities and nuances of social comparison influencing patient perceptions of their diabetes and related risks and their motivation in improving their glycaemic control [32].*** ***In-depth interviews were conducted between August 2023 and December 2023 at a public primary care clinic (Polyclinic) within a healthcare institution in the Eastern region of Singapore providing ambulatory primary care to over 200,000 patients with T2DM.’***

Reference #32 is newly cited in the revised manuscript as:

32. Lim WM. What is qualitative research? An overview and guidelines. Australasian Marketing Journal (AMJ). 2024;0(0). doi:10.1177/14413582241264619

Thank you for suggesting to cite the article by Mubarak et al. describing a qualitative study on interprofessional collaboration between community pharmacists and general practitioners. While we acknowledge the importance of interprofessional collaboration in today’s healthcare landscape, we think this is largely a different context from patient social comparison.

Instead, we have used reference #32 which outlined the complexity of social phenomena and limitations of capturing nuanced experiences numerically, both of which support our rationale for choosing a qualitative design.

5. In the methods, convenience sampling was used, which introduces selection bias. However, this limitation is not explicitly discussed. Acknowledging the potential biases associated with this sampling strategy and discussing its impact on generalizability would enhance the study's transparency.

**Response:**

Thank you for highlighting this. We have described further limitations and biases (changes in bold) in the ‘Discussion’ section:

***‘The study has limitations. Convenience sampling, rather than purposive sampling, was used due to*** ***challenges in recruiting patients into this study, introducing selection bias and limiting generalisability. Relevant attributes such as ethnicity and health literacy may not be adequately represented. Nevertheless, there was a good representation of the participants in terms of age, gender, education level, diabetes duration and control, which provided some degree of variation.*** *The complication prevalence among the SPC should not be interpreted as an individual’s predicted risk of developing complications. For such predictions, validated risk calculators should be used, such as the UK Prospective Diabetes Study (UKPDS) [47] in the UK and Framingham Adult Treatment Panel ATP III [48] in Singapore for cardiovascular disease risk calculation.’*

6. In the methods, the coding process is described, but intercoder reliability is not mentioned. Without this, the rigor of qualitative coding might be questioned. It would be beneficial to report how consistency in coding was ensured, such as through independent coding by multiple researchers or the resolution of discrepancies.

**Response:**

Thank you for highlighting this. We have further described the coding process (changes in bold) in the ‘Data analysis’ section:

*‘RO and KG familiarised themselves with the transcripts from the first two in-depth interviews and independently coded the transcripts. Open codes were assigned to the transcripts based on the study objective, and were combined to form a coding framework.* ***RO and KG met to discuss and reach a consensus on shared meanings of the codes, and discrepancies were resolved through consultation with an experienced qualitative researcher CJN.*** *RO coded the remaining transcripts and new codes were added iteratively after discussion with the study team.*’

7. In the results, the sample size is relatively small (N=18), and the ethnic distribution is skewed toward Chinese participants. However, this is not addressed as a potential limitation. Discussing the impact of this demographic distribution on the transferability of findings would add important context to the results.

**Response:**

Thank you for your feedback. As this qualitative study was embedded within a larger two-arm pilot randomised controlled trial (RCT), the sample size of 12 per arm was determined a priori based on the calculation by Julious. However, with an estimated attrition rate of 50%, a final sample size (N=18, rounded off to 20) was determined in the intervention arm. For this qualitative study, the sample size was guided by thematic saturation, which was achieved after interviewing 12 patients in the intervention arm. A further 6 interviews were conducted to ascertain that no new findings emerged.

The following sentence (in bold) has been added to the ‘Participants and sampling’ subsection of the ‘Methodology’ section:

*‘A sample size of 20 was established based on the recommended sample size of 12 per arm by Julious for feasibility studies without prior information to base the sample size on, accounting for a 50% attrition rate at follow up and missing data [34].* ***For this qualitative study, the sample size was guided by Hennink and Kaiser who found that saturation is often achieved within 9-17 interviews [35].’***

Reference #35 is newly cited in the revised manuscript.

In addition, the following sentence (in bold) has been added to the ‘Data analysis’ section:

*‘Emergent themes were identified.* ***Thematic saturation was reached after 12 interviews. No new themes emerged from the remaining interviews.’***

For the skewed ethnic distribution, we have addressed it as a limitation in the ‘Discussion’ section (in bold):

*‘The study has limitations. Convenience sampling, rather than purposive sampling, was used due to challenges in recruiting patients into this study, introducing selection bias and limiting generalisability.* ***Relevant attributes such as ethnicity and health literacy may not be adequately represented. Nevertheless, there was a good representation of the participants in terms of age, gender, education level, diabetes duration and control, which provided some degree of variation.’***

8. In the discussion, the results are well interpreted, but there is no critical assessment of the potential unintended consequences of the intervention. It would be helpful to consider whether patient similarity-based risk communication could lead to increased anxiety, disengagement, or frustration among individuals with poor control.

**Response:**

Thank you for your feedback. We recognise that patient similarity-based risk communication may have potential unintended consequences and have elaborated on this in the ‘Discussion’ section:

***‘With fear and risk aversity elicited among some participants, the study team recognises the potential for patient similarity-based risk communication to cause increased anxiety, frustration, and possibly disengagement in certain patients. This is consistent with the concerns PCPs raised in the previous study [30] and is a known unintended consequence of risk communication about diabetes complications that could interfere with diabetes self-management [31]. These occurrences can be minimised by PCPs’ awareness of their patients’ contextual factors contributing to glycaemic control, anticipation of negative reactions, and ability to frame and communicate risk information in a person-centric manner while taking the opportunity to motivate patients to improve their glycaemic control.’***

References #30 and #31 are newly cited in the revised manuscript.

9. In the discussion, the authors suggest that social comparison practices need to be stratified, but they do not propose a concrete framework for doing so. Providing a clearer direction on how future studies can operationalize the stratification of patient groups based on comparison tendencies would strengthen this section.

You may consider citing the following study:

DOI: 10.1371/journal.pone.0216563

This study involves expert consensus on structuring collaborative care models, which can be relevant when proposing a structured framework for stratifying patient similarity-based communication approaches.

(This is optional and should only be taken as a suggestion for the improvement of the manuscript.)

**Response:**

Thank you for your feedback. We have described this in the following sentences (in bold) in the ‘Discussion’ session:

***‘Further research is needed to operationalise the stratification of T2DM patients based on their social comparison tendencies and to develop validated measurement tools for data collection. For example, a potential direction is to prospectively study social comparison occurrences, characteristics, and its consequences on T2DM patients practising behavioural change using ‘ecological momentary assessment’ after participants are trained to recognise day-to-day social comparison occurrences. This method intensely captures the nuances of time-sensitive within-person social comparison occurrences and effects over multiple short time periods throughout the day and is less prone to recall bias compared to retrospective self-reporting methods [43].*** ***The findings would shed new insight on how to identify patients who are most*** *receptive to patient similarity-based risk communication and to prioritise its use for these patients, with the aim of advancing their risk awareness and motivating the adoption of protective health behaviours [44].’*

Reference #43 is an existing citation:

43. Arigo D, Mogle JA, Brown MM, Pasko K, Travers L, Sweeder L, et al. Methods to Assess Social Comparison Processes Within Persons in Daily Life: A Scoping Review. Front Psychol. 2020 Jan 22;10:2909. doi: 10.3389/fpsyg.2019.02909. PMID: 32038352; PMCID: PMC6987244.

Thank you for suggesting to cite the article by Mubarak et al. describing a Delphi study to build consensus on collaborative practice between community pharmacists and general practitioners. We think this would be more relevant to interprofessional collaboration than to patient social comparison. Instead, we have cited reference #43 which describes a method to capture social comparison experiences which may guide researchers in future to operationalise the stratification of T2DM patients based on social comparison practices.

10. In the conclusion, it is stated that patient similarity-based risk communication is useful, but there is no critical assessment of who benefits the most from this approach. Clarifying which patient subgroups found this method most effective would make the conclusion more precise and informative.

You may consider citing the following study:

DOI: 10.3389/fpubh.2024.1323102

This study assesses the impact of pharmacist-led interventions on diabetes management, which can support discussions on which patient subgroups benefit most from specific intervention strategies.

(This is optional and should only be taken as a suggestion for the improvement of the manuscript.)

**Response:**

Thank you for your feedback. We have rewritten the ‘Conclusion’ section and it now reads:

***‘This study provided insights into the acceptability of using a social comparison approach in communicating risk to patients with uncontrolled T2DM. It highlights the importance of identifying and selecting patients who are receptive to social comparison, clarifying patients’ perceptions of risks, including zero risk, and providing tailored and socially comparable strategies to mitigate these risks.’***

Thank you for suggesting to cite the article by Iqbal et al. describing a multicentre randomised controlled trial demonstrating greater improvements in HBA1c, blood pressure and cholesterol levels among patients with type 2 diabetes who received pharmacist-led counselling and collaborative prescribing in addition to routine care. Compared to the control group, better medication compliance was reported in the intervention group and a higher proportion of intervention group had their pharmacological therapy escalated to insulin or dual therapy. We think the article would be more relevant for research aiming to identify patient subgroups who would benefit from pharmacist-physician collaboration with a focus on medication counselling and prescribing.

Additional revisions we have made:

1. We have revised the overall manuscript format and file naming according to PLOS ONE’s style requirements. The title, authors and affiliations have been included into the main document.

2. The following have been renamed to appear in the ‘Supporting information’ section:

- ‘S1 Table’ – renamed ***‘S1 Appendix’***
- ‘S2 Table – renamed ***‘S2 Appendix’***
- ‘S3 Table – renamed ***‘S3 Appendix’***

3. The following have been renamed and reformatted to appear in the main article:

- ‘S4 Table’ – renamed ***‘Table 1’***
- ‘S5 Table’ – renamed ***‘Table 2’***
- ‘S1 Fig’ – renamed ***‘Fig 1’***

4. In the ‘Participants and sampling’ sub-section of the ‘Methodology’ section, the following two statements have been added:

***‘None of the participants had a prior relationship with the study team.’***

***‘Of the 93 potential participants approached, 40 consented to participate in the study and 53 declined to participate, with 8 citing unavailability of time or inability to commit, 1 citing inconvenience to participate and 44 citing not being keen or interested in the study.’***

5. In the ‘Patient similarity-based risk communication session and in-depth interview’ sub-section of the ‘Data collection’ section, the time taken for each session has been revised to state a more precise time measurement (changes in bold):

*‘In total, the session and interview took* ***between 38-72 minutes*** *to complete for each participant.’*

6. In the ‘Principal findings’ sub-section of the ‘Results’ section, the description of Theme 3 has been revised (changes in bold):

*‘Several participants* ***expressed concern about developing diabetes complications. They*** *wanted to avoid being like the patients whose HBA1c remained high and developed higher complication rates* ***and this was a wake-up call to improve their diabetes control. Conversely,*** *they aspired to be like the patients whose HBA1c improved over time and developed lower complication rates.’*

Once again, thank you for giving us the opportunity to improve our manuscript. We appreciate the time and effort you have taken to provide valuable feedback to us. We hope that our revised manuscript and the responses we have provided will adequately address all the points raised.

Yours sincerely,

Dr Ruiheng Ong

Family Physician – SingHealth Polyclinics

167 Jalan Bukit Merah, Connection One, Tower 5, #15-10

Singapore 150167

On behalf of:

Professor (Dr) Chirk Jenn Ng

Dr Kalaipriya Gunasekaran

Ms Hang Liu

Professor Wynne Hsu

Professor Mong Li Lee

Associate Professor (Dr) Ngiap Chuan Tan

Correspondence email: ong.ruiheng@singhealth.com.sg
